# Effects of Food-Derived Antioxidant Compounds on In Vitro Heavy Metal Intestinal Bioaccessibility

**DOI:** 10.3390/antiox13050610

**Published:** 2024-05-16

**Authors:** Maria Maisto, Adua Marzocchi, Roberto Ciampaglia, Vincenzo Piccolo, Niloufar Keivani, Vincenzo Summa, Gian Carlo Tenore

**Affiliations:** 1 ChimNutra Labs, Department of Pharmacy, University of Naples Federico II, Via Domenico Montesano 49, 80131 Naples, Italy; adua.marzocchi@unina.it (A.M.); roberto.ciampaglia@unina.it (R.C.); giancarlo.tenore@unina.it (G.C.T.); 2 Department of Pharmacy, University of Naples Federico II, Via Domenico Montesano 49, 80131 Naples, Italy; vincenzo.piccolo3@unina.it (V.P.); niloufar.keivani@unina.it (N.K.); vincenzo.summa@unina.it (V.S.)

**Keywords:** polyphenols, chelation, in vitro digestion, heavy metals, antioxidant activity

## Abstract

Environmental contamination by heavy metals (HMs) has emerged as a significant global issue in recent decades. Among natural substances, food-deriving polyphenols have found a valuable application in chelating therapy, partially limited by their low water solubility. Thus, three different hydroalcoholic extracts titrated in quercetin (QE), ellagic acid (EA), and curcumin (CUR) were formulated using maltodextrins as carriers, achieving a powder with a valuable water solubility (MQE 91.3 ± 1.2%, MEA 93.4 ± 2.1, and MCUR 89.3 ± 2%). Overcoming the problem of water solubility, such formulations were tested in an in vitro simulated gastrointestinal digestion experiment conducted on a water sample with standardized concentrations of the principal HMs. Our results indicate that regarding the nonessential HMs investigated (Pb, Cd, As, Sb, and Hg), MQE has been shown to be the most effective in increasing the HMs’ non-bioaccessible concentration, resulting in concentration increases in Cd of 68.3%, in As of 51.9%, in Hg of 58.9%, in Pb of 271.4, and in Sb of 111.2% (*vs* control, *p* < 0.001) in non-bioaccessible fractions. Regarding the essential HMs, MEA has shown the greatest capability to increase their intestinal bioaccessibility, resulting in +68.5%, +61.1, and +22.3% (*vs* control, *p* < 0.001) increases in Cu, Zn, and Fe, respectively. Finally, considering the strong relation between the antiradical and chelating activities, the radical scavenging potentials of the formulations was assayed in DPPH and ABTS assays.

## 1. Introduction

In recent decades, environmental contamination by heavy metals (HMs) has been significantly increased by anthropogenic emissions, becoming a globally important issue [1]. Specifically, heavy metals and metalloids are elements characterized by an atomic density exceeding 4 g/cm³. This category comprise elements such as copper (Cu), cadmium (Cd), zinc (Zn), lead (Pb), mercury (Hg), arsenic (As), silver (Ag), chromium (Cr), iron (Fe), and platinum (Pt) [2]. These metallic elements are consistently and continuously released into soil, air, and especially, water from different natural and anthropogenic sources [1]. Specifically, water contamination represents HM access throughout the food chain, transferring them to soil, plants, and ultimately, animals. From a biological point of view, heavy metals are not biodegradable [1]. Therefore, the only strategies that the human organism uses to detoxify metal ions are the incorporation of the metal as an active element for the function of physiological enzymes or proteins or their deposition in intracellular insoluble granules that are excreted from the human organism with the feces or stored long-term, determining prolonged storage with dangerous bioaccumulation [3]. It is well known that HM accumulation causes several biological and physiological complications in humans. Although some heavy metals are necessary for several biological functions (e.g., essential elements), prolonged exposure to these elements, even at deficient concentrations, is positively correlated with the occurrence or progression of several diseases, mainly affecting the lungs, kidneys, liver, prostate, esophagus, stomach, and skin [4,5]. In addition, heavy metal exposure is strictly correlated with the development of neurodegenerative disorders, such as Alzheimer’s and Parkinson’s diseases [4,5]. In this scenario, a relevant public issue is the HM human health risk assessment [4,6]. For a predictive and plausible evaluation of the HM health threat, the risk assessment could be conducted considering not only the HM concentration in ingested soil, water, or vegetables but it could be reasonably assessed considering HM bioaccessibility, i.e., the fraction that can be absorbed by a human after HM ingestion.

The main available models used for the assessment of HM bioaccessibility are in vitro experiments, based on the evaluation of gastric and intestinal bioaccessibility, and in vivo techniques by evaluating biological processes such as biomarkers and bioaccumulation. Recently, in vitro models have been preferred due to their advantages of being very cost-effective and especially because they can be performed without any ethical issues [7]. In this regard, several studies have demonstrated that HMs are characterized by a non-negligible intestinal bioaccessibility ranging from 4.2% (Cu) to 64.1% (Pb) [6,8,9,10].

In recent years, several food-derived molecules have shown various health-promoting effects [11,12,13]. In line with this emerging trend, research into natural compounds with chelating properties has caught valuable attention in the area of environmental and health sciences [14].

**Figure 1:**
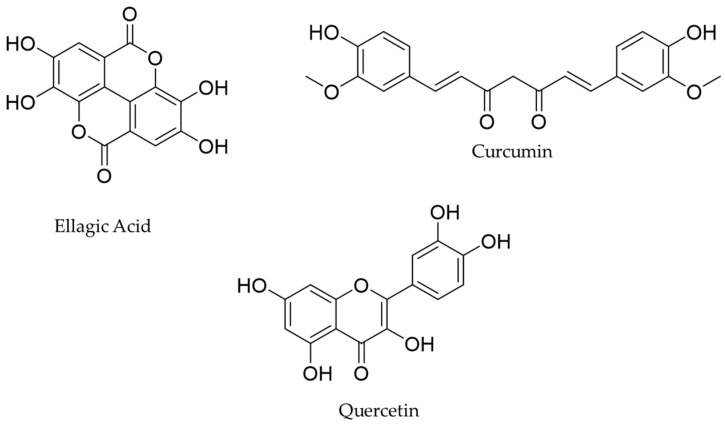
Chemical structure of ellagic acid, curcumin, and quercetin.

Chelation represents the process of forming stable complexes with heavy metal ions and plays a pivotal role in mitigating the adverse effects of heavy metal toxicity in humans. Among the myriad of naturally occurring chelating agents, polyphenols (especially flavones) can successfully chelate metal ions like Al(III), Fe(II), Fe(III), Cu(II), or Zn(II) [14]. Due to their multiple hydroxyl groups, they provide an ideal framework for metal ion coordination. In this regard, quercetin (QE, 3,3′,4′,5,7-pentahydroxyflavone; Figure 1) is a flavonoid found in several fruits and vegetables and is characterized by a well-known antioxidant potential. Beyond its antioxidative effects, recent studies have highlighted its ability to form stable complexes with Cd [15,16]. Likewise, ellagic acid (EA, 1,2,3,4,6-pentahydroxybenzene-3,4,5,6-tetracarboxylic acid; Figure 1), another naturally occurring polyphenol, is mainly contained in berries and pomegranate [17]. Structurally, EA belongs to the class of phenolic acids, deriving from the condensation of gallic acid molecules, and possesses a distinctive chemical structure comprising multiple hydroxyl and carboxylic acid groups. This structural arrangement lends itself to effective metal chelation. Several studies indicate that EA reduces the Pb, Cd, and Hg levels in different study models. Malamacci and colleagues have underlined that EA treatment could reduce the level of Pb, Cd, and Hg in the SH-SY5Y neuroblastoma cell model, underling its potential chelating activity in neuronal diseases [18]. In another experimental model, EA-based treatment resulted in a reduction in kidney Pb concentration in a rat model [19], supporting its metal-chelating potential. In line with this trend, curcumin (CUR; Figure 1), a hydrophobic low molecular weight polyphenolic compound largely used in nutraceutical and cosmeceutical products [20,21,22], has also gained particular attention for its heavy metal-chelating activity [23]. Structurally, the presence of keto-enol tautomeric forms in curcumin enhances its metal-binding capabilities. It was described that due to the proton depletion in the enol form of CUR, the β-diketone part is the main site of chelation for Cu (II), forming a CU-Cu (II) stable complex [24]. The main limitation of the effective usage of these molecules for chelating therapy could be related to their very low water solubility [25,26,27].

In line with these observations, the principal objective of the current study was to test a maltodextrin-based water-soluble formulation of quercetin (MQE), curcumin (MCUR), and ellagic acid (MEA), as well as of their mixed version, in relation to their chelating potential during the gastrointestinal digestion process conducted on a water sample with a standardized HM concentration. The selection of this study model was based on the aim of investigating the chelating activity of selected polyphenols without the interference of food matrices.

## 2. Materials and Methods

### 2.1. Reagents

All of the extracts employed in the current study were supplied and certified by Farmalabor s.r.l. (Canosa, Italy). In particular, all of the plant materials employed are ethanolic extracts differently characterized, including *Punica granatum* L. tried at 70% of ellagic acid (*w*/*w*), *Sophora japonica* L. tried at 95% of quercetin (*w*/*w*), and *Curcuma longa* L. tried at *95%* of curcumin (*w*/*w*). All of these extracts were tested separately or mixed at a ratio of 1:1:1 *w/w* of the bioactive compound.

### 2.2. Encapsulation Process by Lyophilization

Maltodextrins (MDs) used as the encapsulating agent [28] were suspended at a concentration of 20% (*w/v*) in a distilled water solution acidified with 1% citric acid. Then, QE, EA, and CUR, separately and in combination (MIX), were mixed with the MD suspension at a ratio of 1:9 (*v/v*) and left under magnetic agitation (L-81, Labinco, The Netherlands) for 30 min at room temperature. After this time, the different formulations obtained were frozen for 24 h at −40 °C (Coldlab, Brazil) and freeze-dried (LS 6000, Terroni^®^ Equipment, São Carlos, Brazil) for 96 h (−54 °C, 230–300 Hg). After obtaining the dried samples, they were ground into a fine powder using a mortar and pestle, then sifted through a sieve with 32 mesh openings measuring 0.500 mm/m. The powdered samples were then stored in airtight plastic bags placed in desiccators at room temperature until they were needed.

### 2.3. Moisture Content and Water Activity

To determine the moisture content and water activity, 2 g of the powdered samples was accurately weighed and placed in a Petri dish that had been previously dried and weighed. The Petri dish with the samples was then placed in an oven (105 ± 2 °C for 2–3 h) until a constant weight was achieved. The moisture content was determined by comparing the weight before and after drying [29]. The water activity was calculated in triplicate using a Decagon Aqualab Lite (BrasEq-S’eries, 3B).

### 2.4. Hygroscopicity

For hygroscopicity determinations, 1 g of each lyophilized powder was placed into a desiccator with a saturated NaCl (75%) solution for 7 days at 25 °C. After this period, the samples were weighed, and the hygroscopicity was calculated as g of adsorbed moisture per 100 g of dry powder and reported as a percentage [30].

### 2.5. Water Solubility

Solubility evaluation was determined according to a procedure previously described by other authors [31]. In total, 1 g of each formulation and the native extract were suspended in 100 mL of distilled water and left under agitation for 30 min at 110 rpm, followed by centrifugation for 5 min at 4000 rpm. An aliquot of 5 mL was collected from each sample, transferred to a previously weighed Petri dish, and left in a laboratory oven at 105 °C until the reaching of a constant weight. The difference in Petri dish plate weight allows for the calculation of solubility. Solubility was calculated as the percentage of the weight of the dried solution compared to the weight of the sample originally added (1.0 g). We took out samples (5 mL) from each supernatant, moved them to a Petri dish which was weighed beforehand, and dried them until they reached a stable weight in an oven at 105 °C. Then, we calculated the solubility by comparing the weight of the dried solution to the weight of the originally added sample (1.0 g), expressing it as a percentage [32].

### 2.6. Phenolic Compound Encapsulation Efficiency

In order to establish the encapsulation performance of the used protocol, the total phenolic content (TPC) was assessed in both the encapsulated and nonencapsulated fractions. Specifically, the encapsulated phenolic compounds were liberated by the complete destruction of the coating material, following a previously reported procedure [33]. Specifically, 15 mg of all of the formulations was weighed and treated with 3 mL of a solution of ethanol, acetic acid, and water (50:8:42, *v/v*). The mixture obtained was agitated for 1 min using a vortex mixer (Scientific Industries, Bohemia, NY, USA, G-560), followed by 20 min of ultrasound treatment (MaxiClean1650A, Indaiatuba, SP, Brazil) and vacuum filtration using qualitative filter paper (0.45 μm, Millipore filter). The determination of the TPC was performed using the Folin–Ciocalteu method [34] and the results were calculated using a standard curve, expressed in mg of gallic acid equivalent (GAE) per g of dry sample. Encapsulation efficiency was calculated in accordance with a previously described method, with slight modifications [35]. The nonencapsulated phenolic content was extracted from 20 mg of microcapsules in a 1:1 water/ethanol mixture (*v/v*, 1 mL). The solution was vortexed for 10 s, then centrifuged for 10 min at 600× *g*. The supernatant was transferred to flasks and diluted by 10 mL of 40% ethanol. As performed for the encapsulated phenolic fraction, the free phenolic compound amounts were quantified using the Folin–Ciocalteu assay. The encapsulation efficiency (EE%) of the bioactive compounds was determined using the following equation. All of the experiments were performed in triplicate.

$$EE=\frac{\left(1-Superficial\;phenolic\;content\;of\;particles\right)}{Total\;phenolic\;content\;of\;microcapsules}$$

### 2.7. Antiradical Activity of the Maltodextrin-Based Formulations

#### 2.7.1. DPPH^•^ Radical Scavenging Assay

The antioxidant activity of the samples was explored by measuring their capacity to scavenge the stable radical 2,2-diphenyl-1-picrylhydrazyl (DPPH^•^). The assay involved combining 100 µL of each sample, appropriately diluted in an extraction solvent, with 1000 µL of a methanolic solution containing DPPH (153 mmol/L). The mixture was then allowed to incubate for a reaction time of 10 min in the dark. The reduction in absorbance was determined using a UV–visible spectrophotometer (Beckman, Los Angeles, CA, USA). Following the incubation time, absorbance was measured at 517 nm. All determinations were conducted in triplicate. The scavenging of DPPH^•^ was calculated using the following equation:

$$\%\;of\;scavenging=\left(\frac{Ai-Af}{Ac}\right)\;*\;100$$

where Ai represents the absorbance of the sample at t = 0, Af is the absorbance of the sample after the reaction time, and Ac is the absorbance of the control. The control was prepared by mixing 1000 µL of a methanolic solution of DPPH with 100 µL of methanol. The results are generally expressed in µmol of Trolox (6-hydroxy-2,5,7,8-tetramethylchroman-2-carboxylic acid) equivalents (TE). Additionally, the outcomes are calculated as EC_50_, which signifies the quantity of antioxidant compound required to eliminate 50% of the initial DPPH^•^ amount.

#### 2.7.2. ABTS^•^ Radical Scavenging Assay

The ABTS assay is a method for assessing antioxidant activity based on the capability of compounds to react with ABTS^•+^ radical (2,20-azino-bis(3-ethylbenzothiazoline-6-sulfonate)). The experimental procedure followed the protocol previously established by Maisto et al. (2022) [36], with some adjustments. The ABTS solution was prepared by vigorously mixing 2.5 mL of an ethanolic solution containing 7.0 mM ABTS with 44 µL of an aqueous solution of potassium persulfate at a concentration of 140 mM. This mixture was stored in darkness for at least 7 h at 5 °C. Subsequently, a working solution was diluted in the ethanol–water solution until an absorbance value of 0.700 ± 0.05 at 754 nm was obtained (Jasco Inc., Easton, MD, USA). The assay involved combining 1000 µL of the ABTS working solution with 100 µL of the sample, previously diluted in the extraction solvent. After mixing, the solution was kept in the dark for 2.5 min. Following this incubation time, the absorbance of the samples was measured at 734 nm. In the control, the samples were substituted with an equivalent volume of ethanol. The extent of radical inhibition was computed using a specific formula:

$$\%\;of\;scavenging=\left(\frac{Ai-Af}{Ac}\right)\;*\;100$$

where Ai is the absorbance of the sample at t = 0, Af is the absorbance after 2.5 min, and Ac is the absorbance of the control at time zero. Trolox was used as the antioxidant positive control. The results are calculated in both µmol of TE and EC_50_, which represents the amount of antioxidants required to reduce the initial ABTS^•+^ concentration by 50% [37].

### 2.8. Water with Standardized Heavy Metal Concentrations (WSHMC)

A 12.5 mL multielement solution was composed as follows: Cr 50 ng/mL; Ni 50 ng/mL; Hg 100 ng/mL; Pb 100 ng/mL; Cd 10 ng/mL; As 100 ng/mL; Sr 100 ng/mL; Sb 100 ng/mL; Zn 10 ng/mL; Cu 10 ng/mL; Fe 10 ng/mL. To this solution was added 1 g of each maltodextrin-based formulation previously prepared. The samples were left under magnetic stirring until complete powder dissolution.

### 2.9. Bioaccessibility Study of HMs

#### 2.9.1. Simulated Gastrointestinal Digestion of WSHMC

WSHWC with maltodextrin-based formulation prepared was subjected to sequential oral, gastric, and intestinal in vitro digestion, following a harmonized procedure reported by the COST action INFOGEST network. We prepared simulated digestion fluids, such as gastric fluid (SGF), salivary fluid (SSF), and intestinal fluid (SIF), using a method outlined in an earlier procedure [38,39]. Briefly, the formulation was mixed with 3.5 mL of SSF at a temperature of 37 °C. Next, 25 µL of 0.3 M calcium chloride, 0.5 mL of α-amylase solution (75 U/mL), and 975 µL of water were added and mixed. After that, the pH value was regulated at 7 using a 1 M hydrochloride acid (HCl) solution, and the mixture was incubated at 37 °C for 2 min in an orbital shaker bath at 200 rpm. Then, to simulate gastric conditions, 5 µL of 0.3 M calcium chloride, 7.5 mL of SGF, 1.6 mL of pepsin solution (2000 U/mL), and 695 µL of water were added and carefully mixed. After that, a solution of 1 M HCl was used to modify the pH of the mixture to 3, which was then incubated for 120 min at 37 °C in an orbital shaker bath at 200 rpm. After this incubation period, to simulate the intestinal phase, 1.3 mL of water, 11 mL of SIF, 2.5 mL of pancreatin solution (100 U/mL of trypsin activity), 5 mL of bile salt solution (65 mg/mL), and 40 µL of 0.3 M calcium chloride were added. After that, the solution was mixed, and 1 M NaOH was added to modify the pH of the mixture to 7. The solution was incubated at 37 °C for 2 h at 200 rpm in an orbital shaker bath. At the end of the incubation, the samples were centrifuged at 9000 rpm for 10 min at 37 °C. The supernatant and the pellets were separately collected and freeze-dried. The powder obtained was stored at −20 °C until the analysis.

#### 2.9.2. Determination of Heavy Metals through Atomic Absorption Spectrophotometry (AAS)

For digestion, the samples were treated with highly pure concentrated nitric acid (65%) and hydrogen peroxide (30%) and digested using a microwave digestion apparatus (MW-AD, Ethos EZ microwave digester, Mileston, Shelton, CT, USA). The samples were placed in TFM^®^PTFE vessels, and 6 mL of concentrated nitric acid (65%) and 1 mL of hydrogen peroxide (30%) were added. The digestion process involved heating at 90 °C for 7 min, followed by 170 °C for 5 min, 210 °C for 5 min, and finally 210 °C for 20 min. The resulting solutions were diluted to 25 mL with distilled water.

Determination of As, Pb, Hg, Cd, Sb, Cu, Zn, and Fe total contents was performed by using an AA-6300 spectrophotometer (Shimadzu, Columbia, MD, USA) furnished with an ASC-6100 autosampler (Shimadzu, Columbia, MD, USA) and a GFA-EX7i graphite furnace atomizer (Shimadzu, Columbia, MD, USA). Multi-Element Program Software 4.3 was used. Argon was employed as the internal and external gas, with a hole cathode lamp for Cr, Se, Cu, Zn, and Mn, a deuterium lamp as a background corrector, and graphite pyrolytically coated tubes. To optimize the analytical signal, various tests with different parameters, such as varying lamp intensities, sample injection volumes, and temperature ranges (1600–1800 °C for atomization), were used. Detection of As, Pb, Hg, Cd, Sb, Cu, Zn, and Fe was performed by using atomic absorption spectrophotometry according to the method (AOAC International, 1995), using an AA-6300 spectrophotometer (Shimadzu, Columbia, MD, USA) equipped with an ASC-6100 autosampler (Shimadzu, Columbia, MD, USA), a GFA-EX7i graphite furnace atomizer (Shimadzu, Columbia, MD, USA), graphite pyrolytically coated tubes, and Multi-Element Program Software. Argon was used as the internal and external gas with a hollow cathode lamp for As, Pb, Hg, Cd, Sb, Cu, Zn, and Fe and a deuterium lamp as a background corrector. The analytical and sensitivity parameters of the method are reported in Table 1.

**Figure 2:**
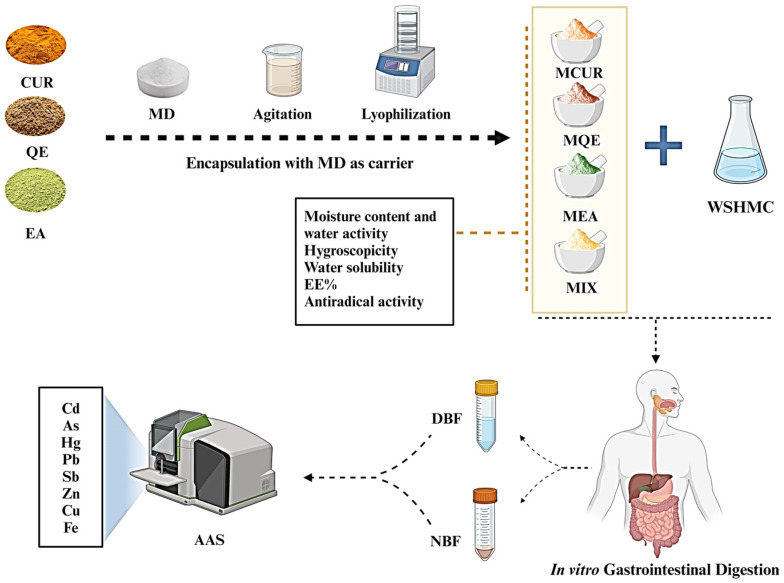
A graphical representation of the experimental model. Abbreviations: CUR, curcumin; QE, quercetin; EA, ellagic acid; MD, maltodextrin; MCUR, maltodextrin-based formulation of curcumin; MQE, maltodextrin-based formulation of quercetin; MEA, maltodextrin-based formulation of ellagic acid; MIX, maltodextrin-based formulation of mixed polyphenols (curcumin, quercetin, and ellagic acid); WSHMC, water with standardized heavy metal concentrations; DBF, duodenal bioaccessible fraction; NBF, non-bioaccessible fraction; AAS, atomic absorption spectrophotometry.

Finally, the experimental design used is illustrated in the following image (Figure 2).

### 2.10. Statistics

Unless specified otherwise, all experimental results are presented as the average value ± standard deviation (SD) from three repetitions. The creation of the graphs and the determination of IC50 values were conducted using GraphPad Prism 8 software. Statistical analysis was performed using SPSS 27.0 software (IBM Corporation, New York, NY, USA), employing one-way analysis of variance (ANOVA) followed by Tukey’s post hoc test. Significance was considered at the confidence level of *p* < 0.001.

## 3. Results and Discussion

### 3.1. Physicochemical Characterization

In order to increase the water solubility of QE, EA, and CUR hydroalcoholic extracts, their encapsulation using MD as the agent was evaluated. At first, the physiochemical characteristics of the powder obtained were investigated, and the observed results in terms of hygroscopicity, water activity (aw), and solubility are reported in Table 1 and Table 2. As expected, a non-significant difference in moisture % was detected between the different formulations evaluated, ranging from 2.1 to 3.6%. These data were in line with those described in the literature, where the moisture content in polyphenolic MD-based formulations obtained by using lyophilization was slightly higher compared to the same formulation prepared through spry-drying because the moisture was largely influenced by the preparation temperature. In the current paper, the studied formulation was prepared by freeze-drying. The choice to use this specific technique is mainly related to its ability to remove moisture from frozen substances through sublimation, working at low temperatures. Consequently, it preserves the chemical structure of thermolabile molecules, such as polyphenols, as well as prevents the generation of impermeable layers, the shift of soluble solids to the surface during drying, and the challenges associated with rehydration [40,41]. Not surprisingly, lyophilization was preferred in several studies over alternative drying techniques. For instance, Rigon and Norena (2015) employed lyophilization to encapsulate anthocyanins extracted from blackberries (*Rubus fruticosus* L.) [42]. Additionally, Khazaei et al. (2014) utilized lyophilization for encapsulating anthocyanins from saffron petals [43], and Saikia et al. (2015) used lyophilization-based encapsulation for the formulation of phenolic compounds extracted from *Averrhoa carambola* L. pomace [44]. Interestingly, the hygroscopicity values follow a different trend compared to the moisture %. Specifically, hygroscopicity refers to the capacity of powders to absorb moisture from the surrounding environment. This characteristic has relevant implications for the physicochemical stability, shelf life, and other various parameters, including the flowability of powders. In the current study, the hygroscopicity of the powders ranged from 20% to 21.6% (Table 2), and the type of extract did not significantly affect the hygroscopicity of the powder obtained. The hygroscopicity capacity was positively influenced by the type and concentration of the encapsulating agents. Considering that the current study employed only a single encapsulating condition, this could explain the non-significant difference between the hygroscopicity value of the prepared preparation. Additionally, our results indicate that higher hygroscopicity values are associated with lower moisture contents. This relationship was explained by a higher water concentration gradient between the powder and the air. In essence, microcapsules showed higher hygroscopicity because they had a smaller amount of humidity [35]. The water solubility of the prepared formulations was between 89 and 93% (Table 3), with the lowest value obtained for the CUR formulation, probably due to its lower water solubility compared to QE and EA. The use of MD as an encapsulant was also related to its well-documented ability to form a more water-soluble formulation compared to other polar encapsulants, such as arabic gum. In particular, several authors have demonstrated the higher solubility of MD-based polyphenol formulations from cherry [45], mango [46], and mountain tea [47] compared to other polar encapsulants. Finally, the last physiochemical parameter analyzed was the EE%, which was a relevant index of the encapsulation process. Our results (Table 4) indicate that the lowest calculated value was obtained in CUR-based powder. This could be related to the evidence that MD was less effective for the encapsulation of lipophilic molecules (LogP: EA 1.05 [48], QE 1.82 [49], and CUR 3.6 [50]). Despite this, our results describe an EE% ranging from 82 to 91%, which is indicative of an efficient encapsulation process. These findings are perfectly in line with the results of a comparative study, where the best EE% of polyphenolic compounds from *Averrhoa carambola* L. was obtained by using MD instead of arabic gum as the encapsulating agent, and lyophilization instead of spray drying as the drying technique [44].

### 3.2. Antiradical Activity of the Polyphenol-Based Formulations

**Figure 3:**
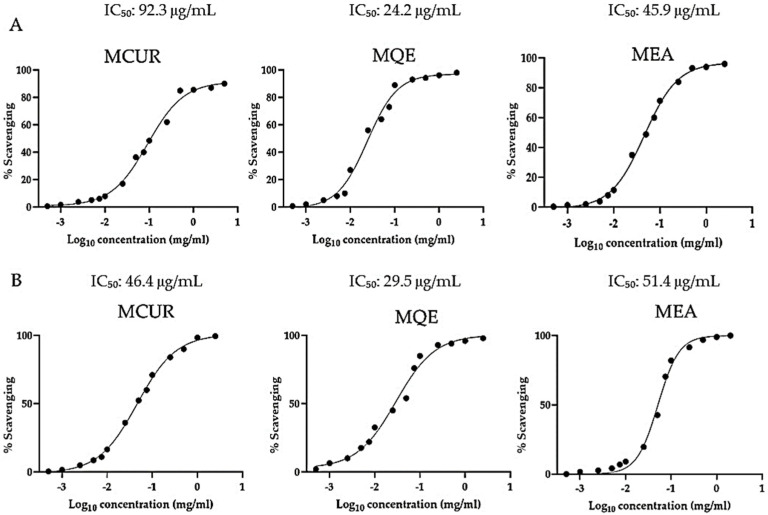
Antiradical activity of MCUR, MQE, and MEA expressed as (**A**) IC_50_ of DPPH assay and (**B**) IC_50_ of ABTS assay. Values represent mean ± standard deviation of triplicate experiment. Abbreviations: MCUR, maltodextrin-based formulation of curcumin; MQE, maltodextrin-based formulation of quercetin; MEA, maltodextrin-based formulation of ellagic acid; IC_50_, 50% inhibitory concentration.

Polyphenols are well-known natural antioxidant compounds. Their strong and valuable antioxidant potential is related to their multiple hydroxyl groups, which provide an ideal framework for radical coordination. Our results show that the QE-based formulation exhibited the highest antiradical activity (with a calculated IC_50_ of 24.14 μg/mL for DPPH and 29.53 μg/mL for the ABTS assay) (Figure 3). MCUR, on the other hand, showed the lowest antiradical activity. This result could be related to their structural differences. In particular, CUR has only four positions useful for the coordination of radicals, compared to EA (four hydroxyl groups and two ketonic functions) and QE (five hydroxyl groups and a single ketonic group). The close relationship between chelating and antioxidant activity is well established.

Specifically, it is well reported that the hydroxylic or carbonylic groups implicated in radical neutralization could additionally complex the metal ions. Particularly, as described for antioxidant potential, it is reasonable that QE could exert the highest chelating activity due its six different metal ion coordination sites, which could differently coordinate metal ions, leading to the formation of different stable complex quercetin ions [51], thus being responsible for its strong chelating potential. Regarding CUR, its keto-enol tautomeric forms are responsible for its metal-binding capabilities; this is related to the proton depletion in the CUR enol form, as the β-diketone part becomes the main point of chelation for metal ions, creating a stable complex [24]. Similarly, for EA, it was reported that its chelating potential is mainly related to seven total coordination positions [52].

### 3.3. Effects of Polyphenol-Based Formulations on Intestinal Bioaccessibility of Toxic HMs

After preparing the three different water-soluble polyphenol-based formulations (MCUR, MQE, and MEA), 1 mg of each of them or their mix was dissolved in 12.5 mL of water with a standardized concentration of HMs in order to evaluate their effects on HM intestinal bioaccessibility during an experiment of in vitro gastrointestinal digestion. To this end, the concentration of the main toxic and essential HMs was evaluated both in the supernatant and pellet fractions, which represent the bioaccessible (duodenal bioaccessible fraction, DBF) and non-bioaccessible fractions (NBF, non-bioaccessible fraction), respectively. According to the results reported in the scientific literature, the bioaccessibility of such metals was drastically decreased during the gastrointestinal process. In this regard, the available data are not homogeneous and consistent due to the influence of several factors on this parameter, including the composition of the food matrix or the pH conditions. In this scenario, our experiments could represent the first attempt to establish the bioaccessibility of essential HMs without the non-controlled interferents of the several components of food matrices. Our results indicate that the addition of a CUR-based formulation could increase the non-bioaccessible fraction in the pellet of cadmium (+25.2% vs control; ns), arsenic (+33.1% vs control; *p* < 0.001), mercury (+11.9% vs control; ns), lead (+263.7% vs control; *p* < 0.001), and antimony (+109.1% vs control; *p* < 0.001). A similar trend was also followed by the treatment containing quercetin, where a statistically significant increase in terms of the non-bioaccessible fraction of the main health-damaging HMs, such as cadmium (+68.3% vs control; *p* < 0.001), arsenic (+51.9% vs control; *p* < 0.001), mercury (+58.9% vs control; *p* < 0.001), lead (+271.4% vs control; *p* < 0.001), and antimony (+111.2% vs control; *p* < 0.001), was obtained. In addition, the solubilization of the EA-based formulation led to a reduction in the intestinal bioaccessibility of the most dangerous HMs, although the effects were less evident in comparison to the other treatments, with a reduced concentration calculated for cadmium (+23.9% in pellet vs control; ns), mercury (+31.9% in pellet vs control; *p* < 0.001), lead (+58.4% in pellet vs control; *p* < 0.001), and antimony (+47.6% in pellet vs control; *p* < 0.001). Considering, instead, the effects of a mixed formulation treatment on HM bioaccessibility, the highest decrease was registered for Pb (with a calculated increase in pellet concentration of +223.9% vs control; *p* < 0.001) followed by antimony (+74.8% in pellet vs control; *p* < 0.001), mercury (+33.9% vs control; *p* < 0.001), and arsenic (+22.5% vs control; ns).

**Figure 4:**
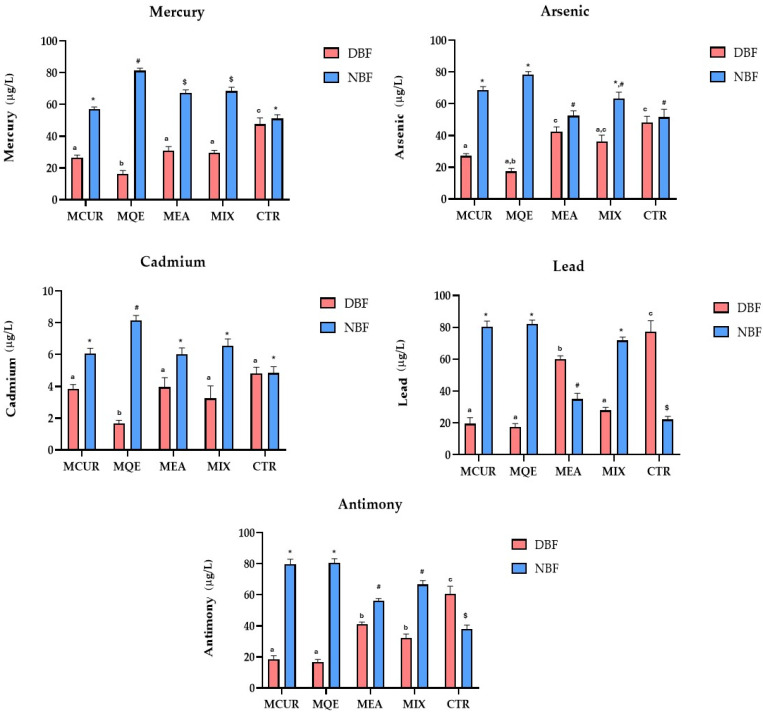
Intestinal bioaccessibility of toxic HMs. Values are presented as mean ± standard deviation of three replicates. Data were analyzed with one-way ANOVA followed by Tukey’s post hoc test; different symbols (*, #, and $) indicate statistically significant differences (*p* < 0.001) between samples in same group (NBF), while different letters (a, b, and c) indicate statistically significant differences (*p* < 0.001) between samples in same group (DBF). Abbreviations: MCUR, maltodextrin-based formulation of curcumin; MQE, maltodextrin-based formulation of quercetin; MEA, maltodextrin-based formulation of ellagic acid; MIX, maltodextrin-based formulation of mixed polyphenols (curcumin, quercetin, and ellagic acid); CTR, control; DBF, duodenal bioaccessible fraction; NBF, non-bioaccessible fraction.

As shown by the above-described results, the QE-based formulation was the most efficient in inducing the precipitation of toxic HMs examined. These data could be related to the higher MQE radical scavenging potential compared to the other formulations studied (Figure 3). Structurally, QE has a dual nature as both an antioxidant and a chelating agent involving intricate molecular interactions that contribute to its therapeutic efficacy. At the heart of its antioxidant activity lies the ability to scavenge reactive oxygen species (ROS) directly, a process driven by the presence of multiple hydroxyl groups in its chemical structure. QE electrons from hydroxylic groups are readily donated to free radicals, neutralizing them and preventing oxidative damage to cellular components. Simultaneously, the QE chelating process involves the formation of coordination complexes with metal ions. This process relies on the availability of an electron-donating hydroxylic group of quercetin polyphenolic structure, allowing it to bind with metal ions through coordination bonds. Thus, QE’s strong antiradical potential could explain the strong and completely unspecific QE-induced HM-chelating potential. In the same way, MCUR and MEA, which are characterized by a valuable antioxidant activity, although lower than that described for QE (IC_50_ of 24.1 μg/mL for DPPH and 29.5 μg/mL for ABTS assay), have shown a chelating potential similar to each other but lower than that of quercetin (Figure 4). Finally, considering the results obtained after the MIX-based treatment, the data indicate that for each HM studied, the chelating rate was second only to the single treatment with QE. This result could be explained by considering that while the mixed formulation is based on equal parts of MCUR, MQE, and MEA, the observed effect is due to the lower active amount of MQE replaced by equal parts of MCUR and MEA, which are, however, characterized by a lower chelating power, as our results show in Figure 4.

### 3.4. Effects of Polyphenol-Based Formulations on Essential HM Bioaccessibility

Considering the essential HMs, our study focused on the evaluation of the effects of the water-soluble polyphenolic formulations on the intestinal bioaccessibility parameters of Zn, Cu, and Fe, high biological impact HMs, due to their well-reported vital roles in biological systems. As described for toxic HMs, for the essential HMs, the available data about their intestinal bioaccessibility are controversial and not homogeneous, drastically influenced by the food matrix composition. Thus, the effects of the MQE, MCU, and MEA-based formulations and their combination on the bioaccessibility of the main essential HMs were investigated. Specifically, our control experiment highlights that the in vitro bioaccessibility of Zn, Cu, and Fe detected was 33.7, 42.1, and 53.3%, respectively. These data partially confirm the scientific evidence available, where the lowest bioaccessibility value was described for Fe. Additionally, Pereira and colleagues have described an in vitro bioaccessibility of Zn as 18%, of Cu as 9%, and of Fe as 41%, in berry samples [53]. Different results were found by Jyothi and colleagues, who reported a calculated bioaccessibility in cashew apple fiber for Zn, Cu, and Fe of 4.0%, 1.2%, and 2.2%, respectively [54]. In contrast, others assessed a Zn and Fe in vitro bioaccessibility of 8.7% and 24.5% in watermelon seed samples [55].

**Figure 5:**
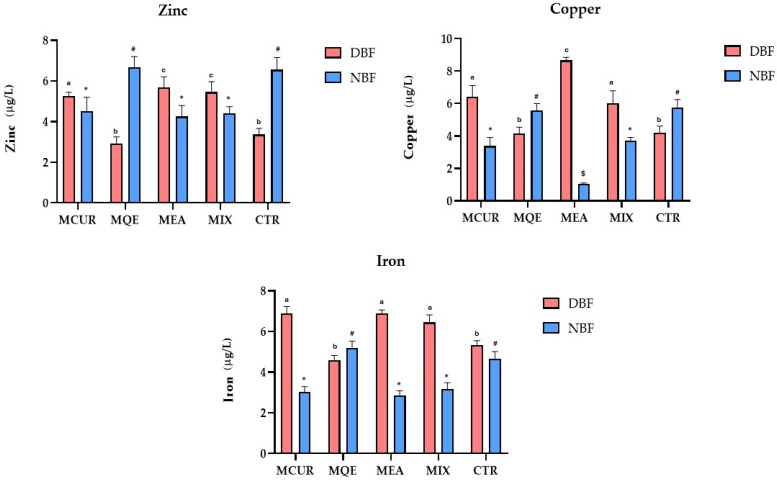
Intestinal bioaccessibility of essential HMs. Values are presented as mean ± standard deviation of three replicates. Data were analyzed with one-way ANOVA followed by Tukey’s post hoc test; different symbols (*, #, and $) indicate statistically significant differences (*p* < 0.001) between samples in same group (NBF), while different letters (a, b, and c) indicate statistically significant differences (*p* < 0.001) between samples in same group (DBF). Abbreviations: MCUR, maltodextrin-based formulation of curcumin; MQE, maltodextrin-based formulation of quercetin; MEA, maltodextrin-based formulation of ellagic acid; MIX, maltodextrin-based formulation of mixed polyphenols (curcumin, quercetin, and ellagic acid); CTR, control; DBF, duodenal bioaccessible fraction; NBF, non-bioaccessible fraction.

In our experimental model, regarding the improvement in Zn bioaccessibility, positive effects were principally observed after the treatment with EA and the mixed formulation, with a significant increase in Zn bioaccessibility (represented by the Zn concentration in the supernatant fraction) of +68.5 and 62.1% vs control (*p* < 0.001) for the EA and mixed treatments, respectively. Additionally, our results indicate that the MCUR treatment led to a small increase in Zn bioaccessibility (+55.8% ppb in the supernatant fraction vs control; *p* < 0.001) and that MQE did not exert any effects on Zn bioaccessibility (Figure 5). Regarding Cu, the MEA treatment led to a valuable gain in its bioaccessibility (+105.6 vs control; *p* < 0.001) in comparison to the increases observed after the MQE, MCUR, and mixed treatments (−1.4, +52.6, and +42.9% vs control; ns, *p* < 0.001) (Figure 5). These results could be explained by considering that on the one hand, the strong MQE antiradical potential makes the coordination with every type of metal ion completely non-selective, leading to their precipitation, whilst on the other hand, the literature largely describes the capability of curcumin to form a stable complex with Cu ions, determining their precipitation [24]. Finally, regarding Fe bioaccessibility, a valuable increase in its supernatant concentrations was obtained after the treatment with all of the formulations assayed (+29.2, +29.4, and +21% vs control, *p* < 0.001, for MCUR, MEA, and mixed treatment, respectively), except for MQE, where a non-significant variation regarding the Fe concentration in both the NBF and DBF was described (Figure 5). Our results indicate that the EA-based treatment was the most effective in increasing Zn and Cu concentrations in bioaccessible fractions. This could be related to EA’s ability to decrease the pH value during the digestion process, creating a soft acidic environment that could positively influence the water solubility of Cu (+36%) and Zn (+19%) [56,57]. Additionally, considering that Cu was characterized by a lower water solubility than Zn, it is reasonable that EA’s pH modulatory effects could be more effective on Cu bioaccessibility. Instead, the effects of the studied formulations on the Fe concentration in the supernatant fraction resulted in an average increase of +10%. This weak but significant data could be explained by considering the antioxidant potential of all of the formulations tested. Practically, the electron-donating potential of MQE, MEA, and MCUR could catalyze the reduction of Fe^3+^ to Fe^2+^, which is a more soluble iron form at the intestinal pH [58]. It is known that at pH values higher than 3, the Fe^3+^ ion forms practically insoluble species. In fact, it was reported that Fe^3+^ bioavailability is very poor just because of its poor solubility at the physiologic pH of the intestine. Finally, regarding the bioaccessibility of such essential elements, the quercetin-based formulation has shown negative effects on the supernatant fraction of all of the essential HMs investigated. This could be related to the fact that QE is a strong chelating agent, with no specific manner of chelation, that could reduce their intestinal bioaccessibility. Regarding the mixed formulation, as shown for the toxic HMs, there were no synergic effects on the essential HMs’ bioaccessibility.

## 4. Conclusions

The valuable results obtained underline that all of the MD-based formulations could be considered polyphenol-based water-soluble formulation prototypes for the development of functional ingredients for nutraceutical formulation with potential chelating activity. All of the formulations prepared using MD as a carrier were characterized by good water solubility and comparable EE%. However, regarding the effect on the bioaccessibility of the heavy metals, the different formulations tested led to completely divergent results. For instance, MQE has shown a strong but also completely non-selective chelating potential, increasing the non-bioaccessible fraction of both toxic and essential HMs, mainly related to its valuable antiradical activity (with a calculated IC_50_ of 24.1 μg/mL for the DPPH assay and 29.5 μg/mL for the ABTS assay). MCUR has demonstrated an effective but less significant chelating potential compared to MEA, as well as the mixed formulation, and has shown no synergic effects on HM bioaccessibility. Interestingly, the surprising results obtained for the bioaccessibility of essential heavy metals could be explained by considering the double potential of the polyphenol-based formulations. In particular, beyond their intrinsic chelating potential related to their peculiar chemical structures, the examined polyphenols, especially EA, could modulate the pH at the intestinal level, which positively influences the solubility of some essential elements, potentially increasing their concentration in bioaccessible fractions. Thus, in conclusion, as a result of our chelating-activity screening, MEA could be a perfect candidate for the development of nutraceutical formulations with HM chelating potential. Indubitably, the formulation process could be optimized in terms of morphological characterization, particle size, and carrier composition. Further studies are likewise necessary to clarify the effective molecular mechanism responsible for such results. It would be advisable to analyze in vitro cellular systems and in vivo animal-based models.

## Figures and Tables

**Table 1:** AAS analytical parameters for heavy metal determination.

Element	Wavelength (nm)	Slit Width (nm)	LR (ppb)	Calibration Curve R^2^	AAS LOD (mg/L)
Hg	253.7	0.2	0–20	0.9819	0.0001
Cd	228.8	0.5	0–10	0.9983	0.0003
As	193.7	0.2	0–20	0.9977	0.0001
Cu	324.8	0.7	0–20	0.9995	0.0001
Pb	283.3	0.2	0–20	0.9991	0.001
Sb	217.6	0.7	0–20	0.9964	0.002
Zn	213.9	30	0–20	0.9988	0.001
Fe	259.94	20	0–20	0.9998	0.001

LR: linear range, LOD: limit of detection.

**Table 2:** Moisture content, hygroscopicity, and water activity of formulated powders.

	Moisture Content %	Hygroscopicity	Water Activity
MQE	3.11 ± 0.02 ^a^	20.3 ± 2.2 ^b^	0.22 ± 0.01 ^c^
MEA	2.11 ± 0.02 ^a^	21.6 ± 3.2 ^b^	0.20 ± 0.01 ^c^
MCUR	3.56 ± 0.02 ^a^	20.0 ± 2.4 ^b^	0.19 ± 0.01 ^c^

Values are presented as mean ± standard deviation of three replicates. Means within same column followed by different letters are significantly different (*p* < 0.005).

**Table 3:** Water solubility of extracts QE, EA, and CUR before and after MD-based encapsulation.

Sample	Solubility (%)
QE	4.12 ± 0.01 ^a^
EA	7.52 ± 0.02 ^b^
CUR	3.21 ± 0.09 ^c^
MQE	91.32 ± 1.23 ^d^
MEA	93.45 ± 2.11 ^d,f^
MCUR	89.32 ± 1.98 ^f^

Values are presented as mean ± standard deviation of three replicates. Means within same column followed by different letters are significantly different (*p* < 0.005).

**Table 4:** Encapsulation efficiency (EE%) of formulations.

Formulation	EE%
MQE	89 ^a^
MEA	91 ^a^
MCUR	82 ^b^

Values are presented as mean ± standard deviation of three replicates. Means within same column followed by different letters are significantly different (*p* < 0.005).

## Data Availability

The data used to support the findings of this study are included in this article.
